# An Unsupervised Tunnel Damage Identification Method Based on Convolutional Variational Auto-Encoder and Wavelet Packet Analysis

**DOI:** 10.3390/s22062412

**Published:** 2022-03-21

**Authors:** Yonglai Zhang, Xiongyao Xie, Hongqiao Li, Biao Zhou

**Affiliations:** 1 School of Civil Engineering, Tongji University, Shanghai 200092, China; 1610227@tongji.edu.cn (Y.Z.); 1410260@tongji.edu.cn (H.L.); zhoubiao@tongji.edu.cn (B.Z.); 2 Key Laboratory of Geotechnical and Underground Engineering of Ministry of Education, School of Civil Engineering, Tongji University, Shanghai 200092, China; 3 China Shipbuilding NDRI Engineering Co., Ltd., Shanghai 200090, China

**Keywords:** in-service train, dynamic response, subway tunnel, damage detection, CVAE, relative entropy, wavelet packet energy, laboratory test

## Abstract

Finding a low-cost and highly efficient method for identifying subway tunnel damage can greatly reduce catastrophic accidents. At present, tunnel health monitoring is mainly based on the observation of apparent diseases and vibration monitoring, which is combined with a manual inspection to perceive the tunnel health status. However, these methods have disadvantages such as high cost, short working time, and low identification efficiency. Thus, in this study, a tunnel damage identification algorithm based on the vibration response of in-service train and WPE-CVAE is proposed, which can automatically identify tunnel damage and give the damage location. The method is an unsupervised novelty detection that requires only sufficient normal data on healthy structure for training. This study introduces the theory and implementation process of this method in detail. Through laboratory model tests, the damage of the void behind the tunnel wall is designed to verify the performance of the algorithm. In the test case, the proposed method achieves the damage identification performance with a 96.25% recall rate, 86.75% hit rate, and 91.5% accuracy. Furthermore, compared with the other unsupervised methods, the method performance and noise immunity are better than others, so it has a certain practical value.

## 1. Introduction

Subway tunnels are the lifeblood of urban rail transit and are essential for residents to travel. Finding a low-cost and efficient method for identifying structural damage is the key to maintaining the safety of subway tunnels and the premise of ensuring normal urban traffic [1,2]. Therefore, this study proposes a new unsupervised novelty detection method to identify tunnel damage using the dynamic responses of in-service trains collected by onboard sensors.

At present, tunnel health monitoring is mainly based on the observation of apparent diseases such as water leakage, settlement, and deformation, such as high-definition photography, laser scanning, radar, and other technologies, and combined with the inspection to grasp the health status of the tunnel [3]. However, these methods generally have high costs and short operating time (only 3 to 4 h in the morning), making it difficult to meet the increasingly stringent requirements of subway tunnel operation and maintenance [4]. The damage identification method based on structural vibration monitoring can identify non-apparent diseases such as structural stiffness degradation and the void behind the tunnel, but, generally, fixed sensors can only be arranged in the vulnerable part of the tunnel to realize automatic monitoring of the tunnel [5]. Because the subway tunnel is deeply buried underground and the stratum parameters are complex, this method lacks effective damage indicators and is expensive. Therefore, there are still many difficulties in application [4]. 

Given the limitations of the existing health monitoring technology, Yang et al. [6,7,8] proposed a method to obtain the bridge vibration mode and identify the structural damage by using the dynamic response of the vehicle when driving on the bridge, which provides a new idea for structural health monitoring. This method has significant advantages such as high mobility, high efficiency, low cost, and continuous testing, and does not require road closures and standstill operations [9]. Hence, vehicle-mounted vibration-based track condition monitoring is widely used in railway track and track bed detection, tunnel health monitoring, and other fields [10,11,12]. Sweden has developed a Rolling Stiffness Measuring Vehicle (RSMV) that can measure the dynamic stiffness of railway tracks [10]. Germany has also developed high-speed ICE-S measuring trains that measure track geometry and vehicle dynamic response [11,12]. Xie [13] and Li et al. [4] proposed a novel method to identify the tunnel damage by using the dynamic response of the subway trains in service and proposed a damage indicator based on the wavelet packet energy change rate and the damage indicator based on the spectral kurtosis change rate. They established a simplified one-dimensional numerical model, refined three-dimensional numerical model, and simple laboratory model tests to verify the damage indicators, and discussed the influence of vehicle speed and load. The results show that the proposed damage indicators can effectively locate the damage of the tunnel structure and has good robustness.

Although the damage identification method based on the dynamic response of the moving vehicle can solve the difficulties of the traditional monitoring method, the method is still in the experimental research stage and is not mature. A large amount of the dynamic response data of in-service trains has a low signal-to-noise ratio and strong randomness. In addition, there is also a lack of effective damage indicators. It is difficult to effectively identify tunnel damage using conventional signal processing methods [13].

With the improvement of computer technology on software and hardware, the increase in data storage capacity, and the reduction in cost, data-driven structural health monitoring (SHM) has attracted more and more attention in the engineering field [14,15]. Compared with traditional methods, SHM based on deep learning and data-driven has the following advantages: (1) Automatic damage identification; (2) Joint training of all parameters with high accuracy; (3) It is suitable for large datasets with high efficiency [15]. At present, data-driven SHM is mainly divided into two directions: vision-based and vibration-based SHM [16]. The former is to essentially process the data of structural deformation, such as displacement, crack, corrosion, and other apparent diseases, which are mainly represented by images or videos. The latter uses the vibration response data measured by the accelerometer deployed on the structure to detect internal and invisible damage. Most of the researchers treat the damage detection tasks in a supervised manner, which can be viewed as a pattern recognition problem in nature, using deep learning methods such as CNN [17,18,19] and LSTM [20]. Chen et al. [21] proposed a new pavement transverse crack detection model based on time–frequency analysis and CNN, and a good recognition performance was achieved by analyzing the measured data. This supervised method requires labeled data in all structural health and damage scenarios to learn [17]. However, it is sometimes difficult to obtain data in all possible damage scenarios, which are rare in practical applications [22].

In contrast, unsupervised learning can avoid such shortcomings, including K-means, K-NN, Auto-Encoder, and its improvement [22,23,24]. Chalapathy et al. [22] developed a deep learning technique for novelty detection based on an unsupervised CNN for structural damage about loose bolts on a bridge model. Ma et al. [23] presented a damage detection method based on a variational auto-encoder for representation extraction and applied it to bridge damage identification under a moving vehicle, and its accuracy was proved by the numerical simulation and in-lab experiment [23]. Li et al. [24] proposed an unsupervised damage detection method based on convolutional auto-encoders and conducts field experiments on the PSC-I bridge to test the accuracy and robustness of the method. Cha et al. [25,26] proposed an unsupervised deep learning method for detecting structural damage with a deep auto-encoder with a one-class of support vector machines, which was verified by steel bridge experiments. However, the major disadvantage of these unsupervised deep learning methods is that they remain inferior in identifying the damage type and degree and detecting low levels of damage [27].

To effectively identify tunnel damage from in-service train dynamic response data, a novel unsupervised deep learning damage identification method is proposed in this study. The method uses the CAVE model to identify whether there is damage and then uses the wavelet packet energy relative entropy to obtain the approximate area of the damage location. The novelties and contributions of this study are as follows. 

(1) A convolutional variational autoencoder model (CVAE) based on in-service train dynamic response is developed to detect the tunnel damage with low cost and high efficiency.

(2) A damage-sensitive indicator (*TDI_WPE_*) based on the relative entropy of wavelet packet energy is developed to identify tunnel damage locations.

(3) A laboratory model that can be automatically controlled is designed and the damage of the void behind the tunnel wall is simulated, and a large amount of experimental data is collected to test the algorithm.

(4) By analyzing the experimental data and comparing with other methods, we show that the proposed method has better recognition accuracy and noise immunity.

The remainder of this paper is arranged as follows. Section 2 introduces the unsupervised method of tunnel damage identification. Section 3 introduces the automated model test system and uses the model test data to train the neural network to verify the effectiveness of the proposed method. Section 4 discusses the comparison between different methods and robustness against noise. Eventually, Section 5 presents the conclusion of this study.

## 2. Methodology

### 2.1. Method Overview

**Figure 1:**
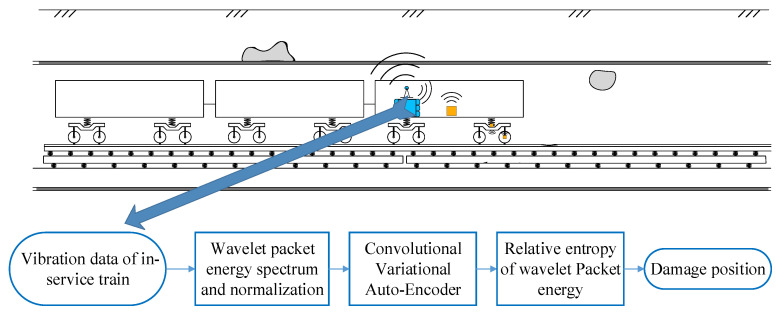
The proposed tunnel damage detection method.

As shown in Figure 1, the tunnel damage identification method uses the onboard accelerometer to collect the vibration response of the in-service subway train and then identifies the damage of the tunnel structure by analyzing acceleration data. The theoretical basis can be found in the literature [4], which has been also verified by numerical simulation and experiments tests.

**Figure 2:**
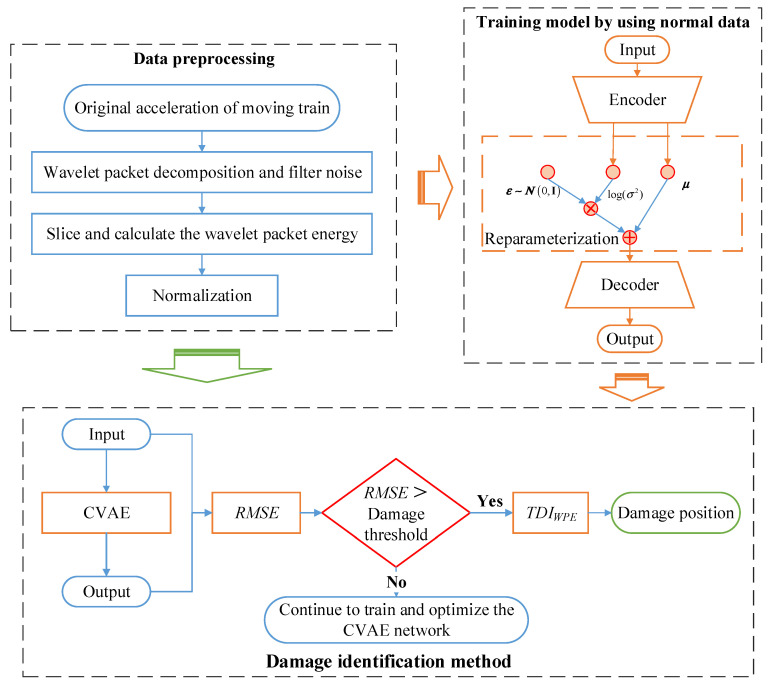
Framework of the proposed tunnel damage detection method.

To monitor the health status of the tunnel in approximately real-time and identify the abnormality of the tunnel when the subway train is in service normally, this study adopts the CVAE model and indicator *TDI_WPE_* to solve the modeling problem. The framework of the proposed damage identification method is shown in Figure 2. The proposed method includes three parts: (1) Raw data preprocessing: After wavelet packet decomposition (WPD) and noise reduction, the wavelet packet energy of each slice is calculated and normalized; (2) Model training: Using the processed data as the learning sample of the model, train and optimize the established CVAE model; (3) Damage identification: The samples are input into the trained CVAE model, and the damage index *TDI_WPE_* of the input and output data is calculated. By comparing the Root Mean Square Error (RMSE) and the damage threshold, it is determined whether there is any damage in the tunnel, and the section where the damage is located is given by *TDI_WPE_*.

### 2.2. CVAE

Variational Auto-encoders (VAE) is a model proposed by Kingma et al. [28], which improves the latent variables of the auto-encoder. The essence of the variational autoencoder is a deep Bayesian network, which is a combination of statistics and neural networks. Compared with Auto-encoders (AE), the core idea of VAE is that the latent variables z are forced to satisfy a specific distribution, such as the standard normal distribution. Considering its independence constraint on latent variables, VAE may be a better feature extraction method.

The CVAE used in this study is to replace the fully connected layer of the VAE with a convolutional layer and a pooling layer, where the encoder and decoder are convolutional neural networks (CNN). In this way, the spatial information of the two-dimensional input data can be preserved, and the network learning ability and computational efficiency can be greatly improved.

**Figure 3:**
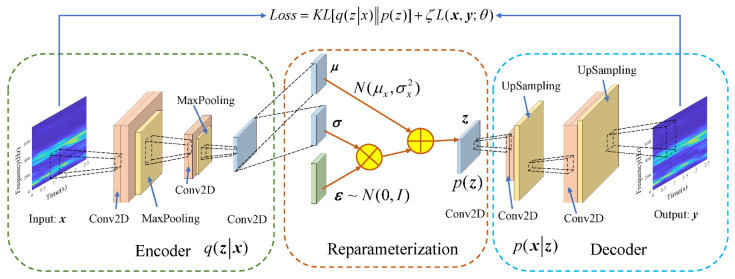
Architecture of CVAE model.

Figure 3 shows the architecture diagram of the CVAE model and shows the expression of the loss function. Conv2D denotes a two-dimensional convolution layer. The two parameter vectors μ and σ, represent the mean and variance of the hidden variable, respectively, so that each element of the hidden variable obeys the corresponding normal distribution $N(\mu_{x},\sigma_{x}^{2})$. The network adopts the technique of resampling and introduces Gaussian noise ε~N(0,I). 

The latent variables z is obtained by:

(1) z=μ+σ⊙ε

where ⊙ represents the Hadamard operator, which represents element-wise multiplication.

The goal of CVAE is to maximize the evidence lower bound (ELBO), which can be expressed as:

(2) $$ELBO=E_{z\sim q(z|x)}[\log p(x|z)]-KL[q(z|x)\|p(z)]$$

where $E_{z\sim q(z|x)}\left[\right]$ is a mathematical expectation calculator; KL[] represents the Kullback–Leibler (KL) divergence that measures the difference between two distributions. For more details on ELBO, please refer to the literature [29].

After simplification, the loss function of the network can be expressed as the following equation [28].

(3) $$L^{*}(\theta )=KL[q(z|x)\|p(z)]+\zeta L(x,y;\theta )$$

The loss function contains two parts that are reconciled by the hyperparameter ζ. The first part is the hidden layer loss, which represents the difference between the predicted distribution q(z|x) of the encoder output hidden variable and the actual prior distribution p(z), which can be represented by KL divergence. After simplification, KL is calculated as follows:

(4) $$\begin{array}{l} KL[q(z|x)\|p(z)]=\int \left(q(z|x)\log\frac{q(z|x)}{p(z)}\right)dz\\ =\int (q(z|x)\log q(z|x)-q(z|x)\log p(z))dz\\ =\frac{1}{2}\sum_{i=1}^{N}\left(\sigma_{i}^{2}+\mu_{i}^{2}-1-\log\sigma_{i}^{2}\right) \end{array}$$

The second part is the reconstruction loss, which represents the difference between the input data and the output data. Generally, the Root Mean Square Error (RMSE) is used, which is calculated according to Formula (5).

(5) $$RMSE=\sqrt{\frac{1}{n}\sum_{i=1}^{n}{\left(y_{i}-{\hat{y}}_{i}\right)}^{2}}$$

To assess whether the data contain anomalies, a damage threshold is set as the criterion. When the RMSE is greater than this threshold, the damage is considered to be present. According to the research of Ren et al., it is assumed that RMSE obeys the normal distribution *N*(*μ_r_*, *σ_r_*), and the upper confidence limit is introduced as the damage threshold. The calculation formula is as follows:

(6) $$L_{1-\alpha }=\mu_{r}+Z_{1-\alpha }\sigma_{r}$$

where *L*_1−*α*_ represents the upper confidence limit, *μ_r_* and *σ_r_* represent the mean and standard deviation of the reconstruction loss RMSE, respectively. The confidence level of *Z*_1−*α*_ is the upper limit of the one-sided confidence interval of 1−*α*. From statistical knowledge, *Z*_1−*α*_ can be calculated as follows:

(7) $$Z_{1-\alpha }=\frac{t_{1-\alpha }(n-1)}{\sqrt{n}}$$

In this model, *α* = 0.05 is preferable, which means that the RMSE is within the one-sided 95% confidence interval (where the RMSE is non-negative), and the sample data can be considered to be normal. Otherwise, the data can be considered abnormal, indicating that the tunnel may be damaged.

### 2.3. Relative Entropy of Wavelet Packet Energy

To identify the location of tunnel damage, a damage indicator *TDI_WPE_* based on relative entropy of wavelet packet energy is proposed in this study. The raw signal is divided into several slices, and *TDI_WPE_* between the input and output of the CVAE in each slice is calculated to determine whether the slice signal is abnormal, and then we will obtain the damage location.

The indicator calculation process is shown in Figure 2. The raw signal x(t) is firstly decomposed by the *j*-layer wavelet packet, and the wavelet packet coefficient $d_{j,k}(t)$ of the *j*-th layer can be expressed by Equation (8). Noise can also be filtered by discarding wavelet coefficients of some frequency bands.

(8) $$D=\left\{d_{j,k}(t),k=1,2,\ldots ,2^{j},j=1,2,\ldots N\right\}$$

Then, the wavelet coefficients are divided into equal-length segment slices, each slice has a length of 𝑙 sampling points, and the translation length is (slice overlap length), then the wavelet coefficients $d_{i,j,k}(t)$ of each slice are expressed as follow:

(9) $$D=\left\{d_{i,j,k}(t),k=1,2,\ldots ,2^{j},j=1,2,\ldots N,i=1,2,\ldots M\right\}$$

The energy spectrum based on the wavelet packet in each slice can be obtained, and it is normalized as a vector:

(10) $$P_{i,j}=\left[\begin{array}{cccc} p_{i,j,1} & p_{i,j,2} & \cdots & p_{i,j,2^{j}} \end{array}\right]$$

where $p_{i,j,k}$ represents the ratio of the wavelet energy of the *k*-th frequency band of the *i*-th slice to the total energy of this slice, which can be calculated as follows:

(11) $$p_{i,j,k}=\frac{e_{i,j,k}}{E_{i,j}}=\frac{e_{i,j,k}}{\sum_{k=1}^{2^{j}}e_{i,j,k}}$$

(12) $$e_{i,j,k}=\sum_{t=(i-1)\times l_{inc}+1}^{(i-1)\times l_{inc}+l}{\left|d_{i,j,k}(t)\right|}^{2}$$

Then, the indicator *TDI_WPE_* of the *i*-th slice can be defined as Equation (13).

(13) $$TDI_{WPE}(i)=\sum_{k=1}^{2^{j}}p_{i,j,k}^{D}\ln(\frac{p_{i,j,k}^{D}}{p_{i,j,k}^{H}})$$

where the superscript *D* represents the train vibration response on the tunnel damage, and *H* represents the signal on the tunnel healthy.

Finally, according to the train speed, the slices are corresponding to the sections of the tunnel space domain.

(14) S=v×t

where *S* is a distance of train running; *v* represents train speed, and *t* represents time.

In each slice, the *TDI_WPE_*(*i*) can measure the difference between the energy distribution of the signal on healthy and damage scenarios. The closer the energy distribution is, the more the relative entropy tends to 0, and vice versa. Therefore, the *TDI_WPE_* can reflect whether the signal is abnormal, and then can find out which slice the damage is in.

To facilitate the quantification of the damage indicator, it is defined that the slice whose *TDI_WPE_* is greater than 20% of the average value of each slice in the sample, may have “damage”, and other slices are considered healthy.

## 3. Experimental Validations

To validate the proposed tunnel damage identification method, model experiments were performed in the laboratory to obtain experimental datasets for method training and performance evaluation. The model test was conducted to verify the feasibility of the tunnel damage detection method based on the vibration response of the in-service train. Furthermore, it was expected that the effectiveness of the proposed method in processing monitoring data would be verified. Due to the limited experimental conditions, we simplified the model significantly.

### 3.1. Experimental Test

#### 3.1.1. Laboratory Physical Modeling System

**Figure 4:**
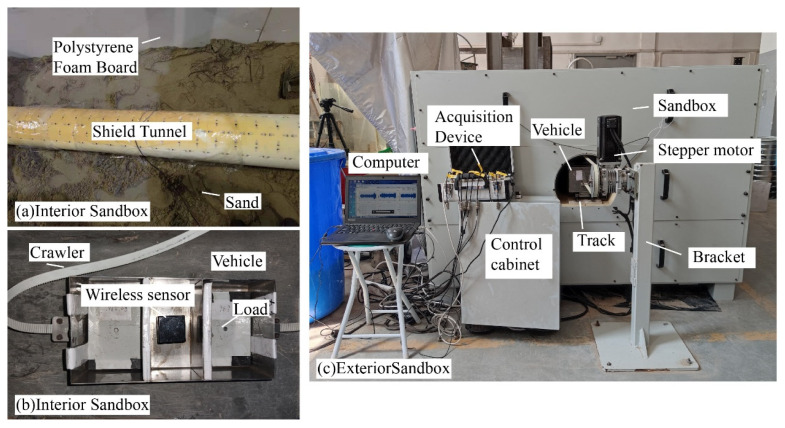
The model and the test system in the laboratory.

Considering the laboratory space, prototype size (taking Jinan subway tunnel as the prototype), model manufacture, and other factors, it was assumed that the geometric similarity coefficient *C_l_* = 1/30, the elastic modulus coefficient *C_E_* = 1/30, and the acceleration similarity coefficient was 1. At the same time, the model did not consider the effects of primary and secondary springs of the train, track fasteners, and groundwater. The structure and field photos of the test system are shown in Figure 4, including the sand box, tunnel, vehicle, power traction, automatic control, and data acquisition system. The key parameters of the model are shown in Table 1.

**Figure 5:**
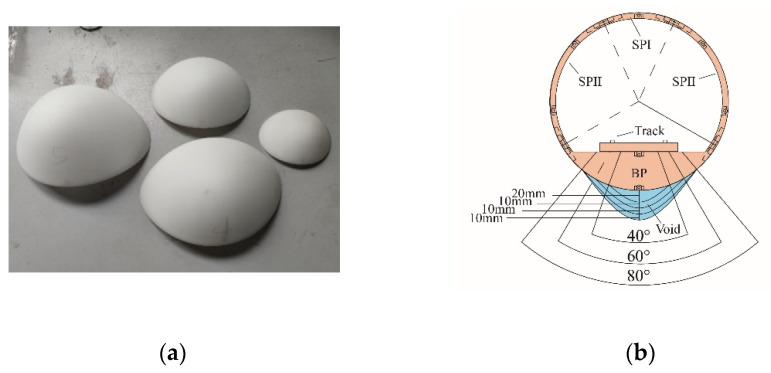
The shape and placement of the voids: (**a**) 3D-printed void, (**b**) void size and position.

The tunnel structure and track plates are fabricated using photosensitive resin 3D printing technology. The outer diameter of the actual tunnel is 6.2 m, and the lining thickness is 0.3 m. Bolts were used to assemble the lining segments, including one standard part-I (SPI, arc 48° × width 10 cm), two standard parts-II (SPII, arc 96° × width 10cm), and a bottom part (BP), as shown in Figure 5. The track is made of two smooth metal strips, reducing the effects of track irregularities.

The sandbox was made of steel. The inner walls of the sandbox were covered with 25 cm thick polystyrene foam boards to absorb vibrational waves and prevent wave reflections that affect the vibrational modes of the structure. Although sand cannot accurately represent the complex real ground, it is useful for controlling boundary conditions and repeatable tests. The sand was compacted during the experiment.

Regardless of the primary and secondary suspension springs of the train, the vehicle was divided into three compartments, which were equipped with wireless sensors and mass. The wireless sensor was fastened with bolts in the middle of the vehicle to collect the vertical acceleration. The sensor sampling frequency was 4000 Hz, with the resolution being 0.2 μg, the measuring range 2 g, and the weight 136 g. Under standard conditions, the vehicle speed and total mass (load and sensor) were 0.9 m/s and 5.08 kg. A stepper motor and tracks pulled the vehicle reciprocating at a constant speed on the track on the track. The test system was automatically controlled by a program to ensure uniform vehicle speed. 

#### 3.1.2. Damage Setup

The tunnel damage was caused by structural material degradation and the external environment. Concrete carbonation, erosion, and spalling reduce lining stiffness, causing tunnel deformation and cracking, leading to water leakage. Soil movement, soil erosion, and surface overloading lead to tunnel subsidence and dislocation, which can lead to tunnel damage.

As shown in Figure 5, the 3D-printed cavity is a parabolic hollow shell of different sizes made of photosensitive resin, which was placed in the soil behind the bottom of the tunnel to simulate the effect of different volumes of the cavity. The void size and position on the tunnel are shown in Figure 5b. To reduce the test workload, four damage levels were set, and the volumes were 566 cm^3^, 331.7 cm^3^, 447 cm^3^, and 166.4 cm^3^, respectively.

#### 3.1.3. Test Conditions

**Figure 6:**
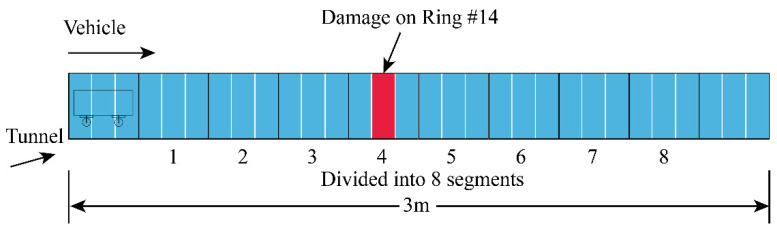
Damage Setup.

Due to the limitations of test materials and equipment, the standard working condition was set as the healthy working condition, with the train speed being 0.9 m/s, and the total mass of the vehicle, load, and sensor being 5.08 kg. In each case, the vehicle reciprocated runs in the tunnel, taking a single trip (from left to right) as a valid sample, and each sample was about 3 s long. As shown in Figure 6, the total length of the tunnel was 3 m, with 30 rings. Among them, 24 rings were analyzed as the effective length and divided into 8 segments. Then, the void damage was placed on the 14th ring, which is on the 4th segment. The test conditions are shown in Table 2. Lastly, 2000 samples were collected repeatedly in Case 0, and 500 samples in each of the other four damage cases.

### 3.2. Data Preprocessing

**Figure 7:**
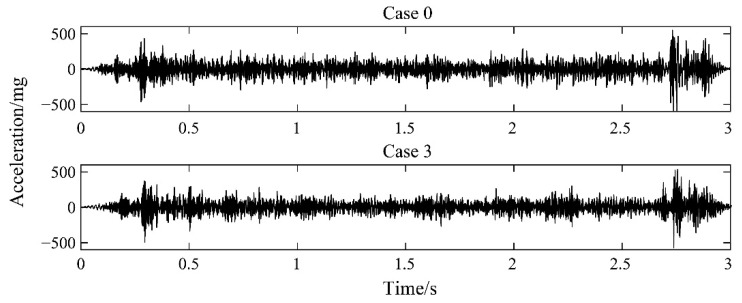
Original signal of the vehicle in modelling test.

Figure 7 shows the collected vehicle’s vertical acceleration of Case 0 and 3. However, it is difficult to directly identify the damage through the time domain signal because of noise. Therefore, we used deep learning methods to solve this problem. According to the method described in Section 2.3, the original data were converted into a WPE distribution map by using the wavelet packet analysis, which was used as the input sample of the deep learning network. The *sym*4 wavelet basis function was used, and the number of decomposition layers was 8. 

**Figure 8:**
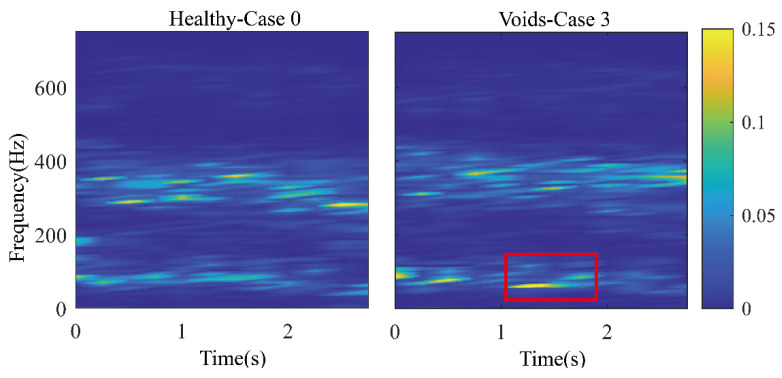
Wavelet packet energy spectrum.

The calculation result is shown in Figure 8. The x-axis in the figure represents the time, and the time window length was 0.25 s, indicating that the time resolution was 0.25 s. The y-axis represents the frequency with a frequency resolution of 7.8 Hz. Each point in the figure represents the ratio of the energy of this frequency band to the sum of the energy of all frequency bands in this time period. The signal energy was mainly concentrated in two frequency ranges, 35.16–144.5 Hz and 261.7–425.8 Hz. Comparing the images of Case 0 and 3, certain subtle differences are observed in the red box in the time period of 1~1.5 s and frequency range of 35.16–144.5 Hz, indicating potential damage in this area. However, other frequency bands are still very noisy, which can affect the damage identification.

As an unsupervised model, the training set samples only selected the test data without tunnel damage and used the K-fold verification method to train the neural network. The partition of the dataset is shown in Table 3. 

### 3.3. CVAE Model Establishing and Training

To select the optimal parameters of the model, first, some initial parameters were set as reference models based on experience, and then the grid search method was used for optimization. The optimized network model structure and parameters are shown in Table 4. The input layer of the model is the normalized WPE distribution map of the train acceleration response data. The size of the input layer is a tensor of 1@18 × 96, where 1 represents one channel, and 18 × 96 represents the size of the map. The algorithm was implemented using Python3.9 and TensorFlow 2.5 and trained on a computer with NVIDIA GTX 1060(GPU) and Intel i7-7700K(CPU). 

### 3.4. Damage Identification

#### 3.4.1. Performance Evaluation Metric

Table 5 presents the confusion matrix for evaluating the detection performance of the method. Based on this matrix, the evaluation metric can be defined as the Equations (15)–(17).

(15) $$Hit\;rate=\frac{TH}{TH+FN}\times 100\%$$

(16) $$Recall\;rate=\frac{TN}{TN+FH}\times 100\%$$

(17) $$Accuracy=\frac{TN+TH}{TN+FN+TH+FN}\times 100\%$$

where the hit rate is calculated as the percentage of void samples correctly detected; recall rate is calculated as the percentage of correctly identified healthy samples; accuracy is a measure of overall performance.

#### 3.4.2. Void Identification

**Figure 9:**
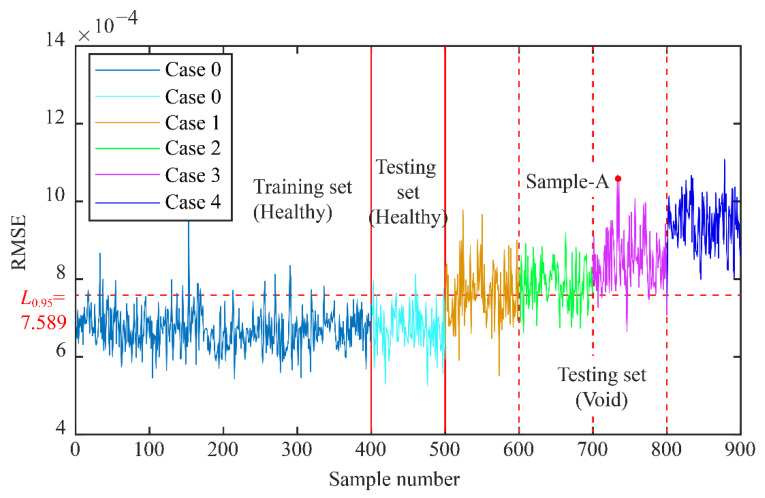
Void identification using CVAE.

Through try and error, hyper-parameters of CVAE were determined using training and validation data. The *RMSE* results of part of the training set and test set are shown in Figure 9. The damage threshold *L*_0.95_ is 7.589 × 10^−4^, which was calculated from the training set results according to Equation (6). When the *RMSE* is less than this threshold, it means that the tunnel is in a normal state, and when it is greater than this threshold, it is considered that the sample is abnormal, that the tunnel may be damaged. The results show that the RMSE of the test set (healthy) is almost all below the damage threshold, and the recall rate is about 96.25%. At the same time, the hit rate is 86.75%, and overall accuracy is 91.5%. As can be seen from the figure, when the void volume behind the tunnel wall is very small, the accuracy of the model to identify abnormalities is low. When the void volume becomes larger, its RMSE value gradually increases and exceeds the damage threshold, and the recognition effect is also significantly improved.

**Figure 10:**
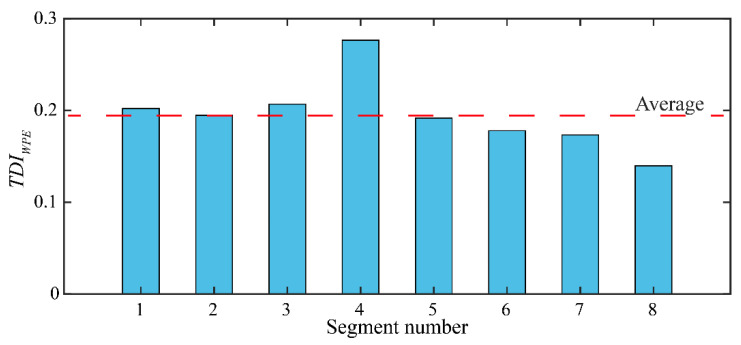
Identify void position using *TDI_WPE_* taking sample-A as an example.

Sample-A is randomly selected from the test set as an example, and the damage location is given by the *TDI_WPE_*. As shown in Figure 10, the *TDI_WPE_* (4) is the largest, which is 0.28, while the average value is 0.196. The results suggest that there may be a void in segment 4. The results of this example verify the effectiveness of the proposed method.

## 4. Discussion

### 4.1. Compared with Other Unsupervised Methods

Experiments show that the method proposed in this study has the advantages of high efficiency, low cost, and strong feasibility. This section compares the proposed method to other unsupervised methods such as Convolutional Auto-Encoder (CAE), Variational Auto-Encoder (VAE), K-means, and Gaussian Mixture Model (GMM). At the same time, the effects of different decomposition methods such as WPD and variational modal decomposition (VMD) are also compared. The identification effects of each model are shown in Table 6.

Comparing the first three methods in the table, the identification accuracy of the model using WPD and VMD methods in each test set is higher than that of the model without any data preprocessing method, and the effect of the WPD-CVAE model is slightly better than that of the VMD-CVAE model. This is because the VMD method only decomposes the vibration amplitude, and the redundant time dimension information is retained, which will generate more random errors when calculating the RMSE, resulting in identification errors. The WPE distribution map obtained by WPD is also compressed in the time dimension, which not only discards some redundant information, but also improves the calculation speed of the model.

When the preprocessing method is WPD, by comparing the identification accuracy of CVAE, CAE, VAE, K-means, and GMM models, it can be found that the CVAE model has the highest identification accuracy in each test set combining the advantages of convolutional layers and variational autoencoders. The conventional K-means and GMM models have low accuracy, and can only identify whether there is an abnormality, but cannot identify the damage location, so they have certain limitations in practical applications. The results show that the proposed method has more superiority for damage detection than other unsupervised methods.

### 4.2. Noise Immunity

**Figure 11:**
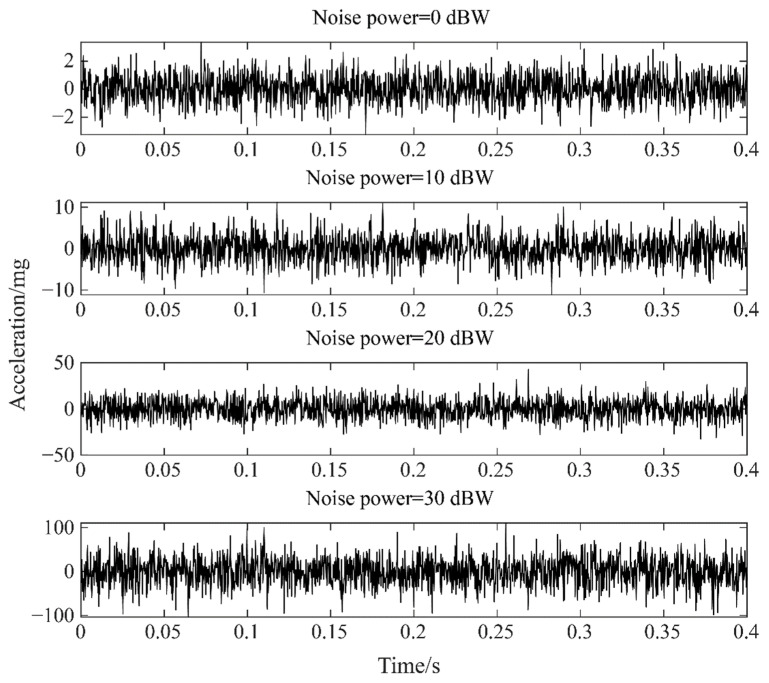
Synthetic WGN of different power.

Due to the influence of the test environment and the measurement error, noise is inevitably generated during the measurement process, which pollutes the original data. So, it will inevitably affect the recognition effect of the detection algorithm. To test the robustness of the proposed algorithm, White Gaussian Noise (WGN) in dBW of different power is injected into the raw data [30]. Figure 11 shows the GWN of 0, 10, 20, and 30 dBW, respectively.

As shown in Table 7, taking Case 3 as an example, the hit rate, recall rate, and accuracy rate of both methods decrease with the increase in noise power. In addition, the results also show that the noise immunity of WPD-CVAE is better than that of VMD-CVAE.

## 5. Conclusions

In this study, we propose a novel automatic detection algorithm for damage localization based on the dynamic response of in-service trains, which has the advantages of high efficiency, low cost, and strong practicability. The proposed method uses CVAE to extract damage-sensitive features, determines whether there is damage, and then uses *TDI_WPE_* to identify the location of the damage. Then, the accuracy and reliability of the proposed method are verified by laboratory tests of the vehicle–track–tunnel–soil coupled vibration model. Finally, the effects of different damage identification methods and noise levels are compared. The following results were obtained:

(1) The proposed method is completely unsupervised and does not require data under various damage scenarios to train the network, which is generally difficult to obtain in practical applications.

(2) An automatic control test system was designed, and the four levels of the void behind the tunnel wall was simulated. The effectiveness of the method is validated by analyzing more than 4500 data samples collected automatically. In the test case, the proposed method achieves a better void identification performance than other unsupervised methods with a 96.25% recall rate, 86.75% hit rate, and 91.5% accuracy.

(3) Compared with VMD-CVAE model, the proposed method fully utilizes the advantages of WPD, CNN, and VAE methods, with higher accuracy and stronger noise immunity.

However, the main disadvantage of this unsupervised method is the inability to identify the damage type and accurately assess the damage extent. Moreover, because the tunnel structure is deeply buried in the ground, the stratum is complex, and the signal-to-noise ratio is low, the quality and quantity of the data will also greatly affect the recognition effect of the model.

Therefore, future work needs to study supervised learning methods that make the best of the manual label of damage information, which can automatically identify the damage type and degree. At the same time, it is necessary to verify the effectiveness of the proposed method through field tests, and accumulate huge amounts and high-quality monitoring data for further research work.

## Figures and Tables

**Table 1:** Key physical parameters of the model.

Components	Parameters	Value
Sand	Depth (m)	0.9
	Density ($\mathrm{kg}\cdot m^{-3}$)	1510
Tunnel	External diameter (m)	0.3
	Length (m)	3
	Depth of tunnel (m)	0.2
	Ring width (m)	0.1
	Lining thickness (m)	0.01
	Density of photosensitive resin ($\mathrm{kg}\cdot m^{-3}$)	1150
	Tensile modulus (MPa)	2779
	Poisson’s ratio	0.42
Sandbox	Length × width × height (m)	3 × 1.5 × 1
Polystyrene foam board	Thickness (m)	0.25
Vehicle	Length × width × height (m)	0.3 × 0.15 × 0.12
	Speed ($m\cdot s^{-1}$)	0.9
	Weight (kg)	5.08
Track	Length × width × height (m)	3 × 0.004 × 0.004
Sensor	Length × width × height (m)	0.04 × 0.04 × 0.05
	Measuring range (g)	2
	Sampling frequency (Hz)	4000
	Weight (kg)	0.136
	Resolution (μg)	0.2

**Table 2:** Test conditions.

Case	0	1	2	3	4
Void/cm^3^	-	166.4	331.7	447	566

**Table 3:** Datasets partition.

Datasets	Condition	Case	Number of Testing Sets
Training	Healthy	0	1280
Validation	Healthy	0	320
Test	Healthy	0	400
	Voids	1	100
	Voids	2	100
	Voids	3	100
	Voids	4	100

**Table 4:** Parameters of CVAE.

Blocks	Layers	Parameters	Output
Encoder	Input	1@18 × 96	1@18 × 96
	Conv2D	32@3 × 3	32@18 × 96
	Conv2D	32@3 × 3	32@18 × 96
	MaxPooling	3 × 3	3 × 3
	Conv2D	16@3 × 3	16@6 × 32
Parameter resampling	*σ* (Conv2D)	4@3 × 3	4@6 × 32
	*μ* (Conv2D)	4@3 × 3	4@6 × 32
	Hidden = *μ* + *σ* × ε	σ_ε_ = 0.01	σ_ε_ = 0.01
Decoder	Conv2D	16@3 × 3	16@6 × 32
	UpSampling	3 × 3	3 × 3
	Conv2D	4@3 × 3	4@18 × 96
	Conv2D	4@3 × 3	4@18 × 96
	Output (Conv2D)	1@3 × 3	1@18 × 96
	Regularization	L2 (1 × 10^−6^)	
	Optimizer	Nadam	
	Batchs	24	
	Epochs	150	

**Table 5:** The confusion matrix.

Sample Number		Prediction	
		Normal	Void
Ground truth	Normal	True normal (*TN*)	False hit (*FH*)
	Void	False normal (*FN*)	True hit (*TH*)

**Table 6:** Testing accuracy of different methods (%).

Data Decomposition Methods in Preprocessing	Machine Learning Methods	Recall Rate	Hit Rate	Accuracy
WPD	CVAE	96.25	86.75	91.5
VMD	CVAE	92.75	84.25	89
	CVAE	88.75	81.5	85.13
WPD	VAE	90.5	82.25	86.38
WPD	CAE	85.25	80.75	83
WPD	K-means	78	78.75	78.38
WPD	GMM	82.25	79.5	80.88

**Table 7:** Performance comparison under different noise power (%).

Noise Power (dBW)	WPD-CVAE			VMD-CVAE		
	Recall Rate	Hit Rate	Accuracy	Recall Rate	Hit Rate	Accuracy
0	95.75	84.5	90.13	91.5	83	87.25
10	95.25	83.25	89.25	90.75	81.75	86.25
20	93.75	82.75	88.25	89.5	80.5	85
30	92.25	82	87.13	88	79.25	83.63

## Data Availability

Not applicable.
